# Plant Viral Proteases: Beyond the Role of Peptide Cutters

**DOI:** 10.3389/fpls.2018.00666

**Published:** 2018-05-17

**Authors:** Bernardo Rodamilans, Hongying Shan, Fabio Pasin, Juan Antonio García

**Affiliations:** ^1^Departamento de Genética Molecular de Plantas, Centro Nacional de Biotecnología, Consejo Superior de Investigaciones Científicas, Universidad Autónoma de Madrid, Madrid, Spain; ^2^Agricultural Biotechnology Research Center, Academia Sinica, Taipei, Taiwan

**Keywords:** viral proteases, viral polyprotein, plant viruses, viral replication, virion formation, host range, defense and counterdefense

## Abstract

Almost half of known plant viral species rely on proteolytic cleavages as key co- and post-translational modifications throughout their infection cycle. Most of these viruses encode their own endopeptidases, proteases with high substrate specificity that internally cleave large polyprotein precursors for the release of functional sub-units. Processing of the polyprotein, however, is not an all-or-nothing process in which endopeptidases act as simple peptide cutters. On the contrary, spatial-temporal modulation of these polyprotein cleavage events is crucial for a successful viral infection. In this way, the processing of the polyprotein coordinates viral replication, assembly and movement, and has significant impact on pathogen fitness and virulence. In this mini-review, we give an overview of plant viral proteases emphasizing their importance during viral infections and the varied functionalities that result from their proteolytic activities.

## Introduction

Viruses are the most abundant biological entities in the planet (Suttle, 2007). With the exception of giant viruses (Wilhelm et al., 2017), viruses share a reduced genome size and optimize a confined genetic space utilizing several strategies of alternative protein production (Firth and Brierley, 2012; Miras et al., 2017). One of these strategies commonly employed by viruses is to produce polyproteins that are further processed by proteases into smaller working units. This strategy ensures production of multiple components required for viral infection in a single molecule and at the same time saves space in the genome by using a single set of transcriptional and translational control elements. It also provides the option to yield partially processed protein products with specific activities, and to alter functionality of a particular protein in a controlled manner (Spall et al., 1997; Konvalinka et al., 2015). However, gene expression through polyproteins relies on proteases for its proper functioning and as such, these enzymes play a central role regulating infectivity and the viral cycle.

Since the discovery of tobacco mosaic virus (TMV) in the late 19^th^ century (Zaitlin, 1998), more than 4000 viral species have been assigned and classified in a total of 131 families (ICTV, 2017; Simmonds et al., 2017). Out of these, 27 families and 9 orphan genera include plant-infecting viruses. The largest family of plant viruses is the *Geminiviridae*, whose members carry a single stranded DNA genome. In eukaryotes, RNA viruses account, however, for the majority of the virome diversity (Koonin et al., 2015). The plant virome is dominated by viruses with positive-stranded RNA genomes, which can be further subdivided into superfamilies based on RNA-dependent RNA-polymerase (RdRp) phylogenetic relationships: Alphavirus-like, and Picornavirus-like (Goldbach et al., 1991; Koonin, 1991; Dolja and Koonin, 2011), *Potyviridae* being the largest representative family of the latter class (Ivanov et al., 2014). Among plant viruses there are also pararetroviruses and viruses with negative-stranded and double-stranded RNA genomes.

**FIGURE 1:**
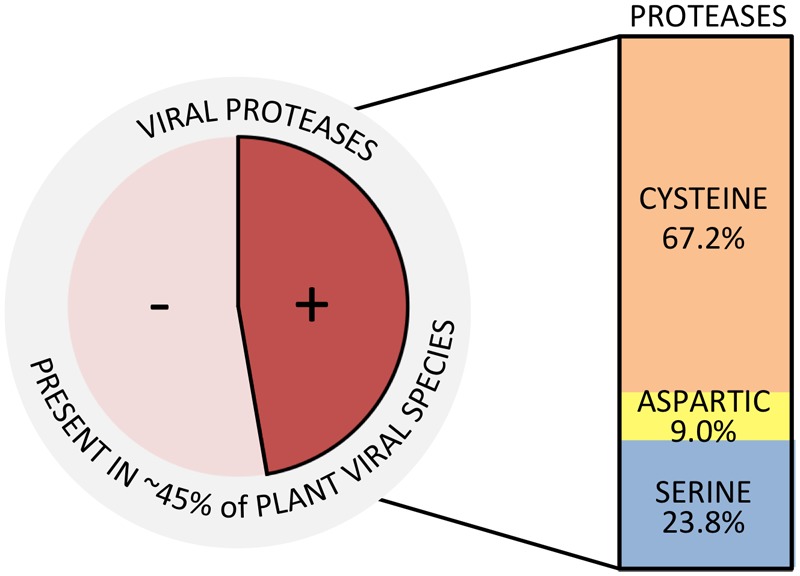
Plant viral species encoding proteases. Total number of plant viral species was based on the ICTV Master Species List (ICTV, 2017). Types of proteases and percentages were calculated counting the total number of proteases present in each of the accounted viral species.

**FIGURE 2:**
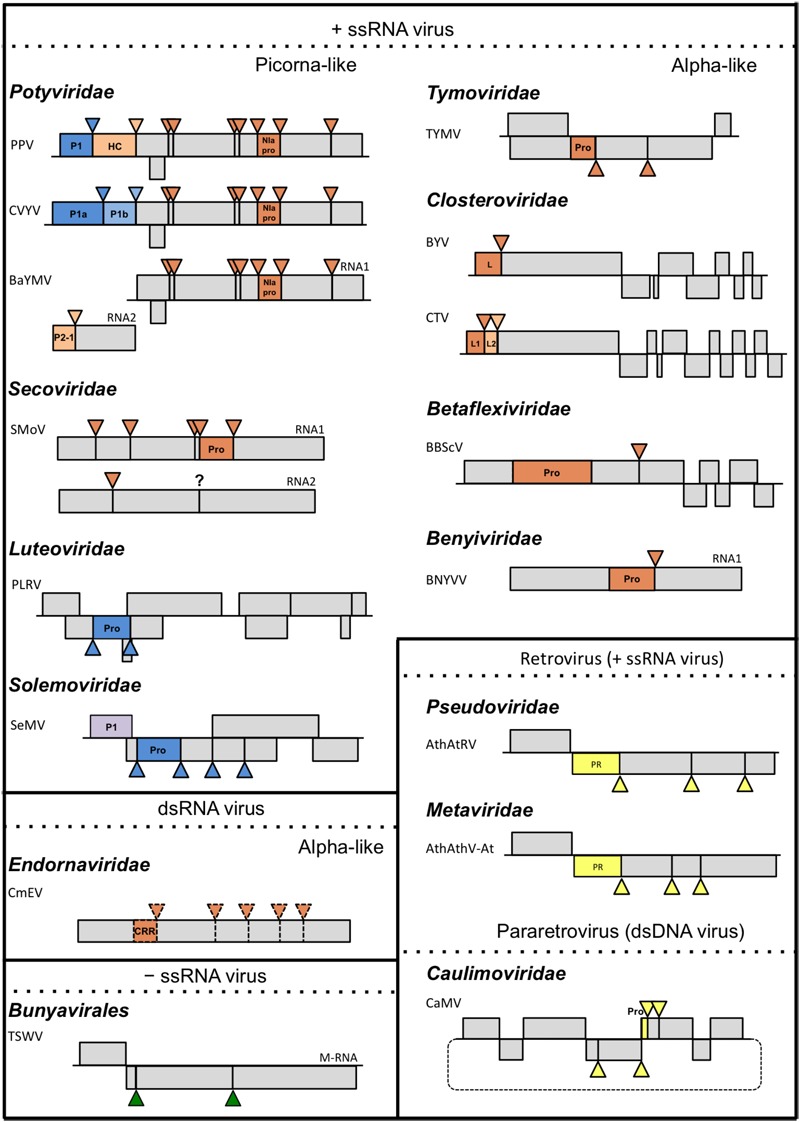
Schematic representation of plant viruses and their proteolytic cleavage sites. Triangles represent cleavage sites of endopeptidases. Colors of the Continuedtriangles match the colors of the corresponding endopeptidases: orange for cysteine, blue for serine, yellow for aspartic, purple for unknown and green for plant proteases; only genomes, or sub-genomes encoding polyproteins subject to proteolytic cleavage are depicted. For each family, a representative species covering the different endopeptidases are depicted. TSWV is included as representative member of the order *Bunyavirales*. Scale of the genome map is maintained only within each viral species. Dotted lines used in the *Endornaviridae* family indicate that processing is only theoretical. Question mark indicates that the way of processing is unknown. PPV, *Plum pox virus, Potyvirus*; CVYV, *Cucumber vein yellowing virus, Ipomovirus*; BaYMV, *Barley yellow mosaic virus, Bymovirus*; SMoV, *Strawberry mottle virus*, unassigned; PLRV, *Potato leafroll virus, Polerovirus*; SeMV, *Sesbania mosaic virus, Sobemovirus*; TYMV, *Turnip yellow mosaic virus, Tymovirus*; BYV, *Beet yellow virus, Closterovirus*; CTV, *Citrus tristeza virus, Closterovirus;* BBScV, *Blueberry scorch virus, Carlavirus*; BNYVV, *Beet necrotic yellow vein viru*s, *Benyvirus*; CeMV, *Cucumis melo alphaendornavirus, Alphaendornavirus*; TSWV, *Tomato spotted wilt orthotospovirus, Orthotospovirus*; AthAtRV, *Arabidopsis thaliana AtRE1 virus, Pseudovirus*; AthAthV-At, *Arabidopsis thaliana Athila virus, Metavirus*; CaMV, *Cauliflower mosaic virus, Caulimovirus*.

Synthesis of viral endopeptidases occurs in ∼45% of plant-infecting species (**Figure 1**), grouped into 12 families (**Figure 2** and **Table 1**) (ICTV, 2017). These viruses encode three types of proteases: cysteine (67.2%), aspartic (9.0%), and serine (23.8%) proteases (**Figure 1**), which belong to 12 catalytic families (**Table 1**), according to the peptidase database MEROPS (Rawlings et al., 2013). Viral endopeptidases share certain features that make them distinct from host proteases: (i) they are smaller, (ii) they present little sequence similarity that might be restricted to active site residues, (iii) they can adapt to multiple roles, and (iv) they are very specific in their cutting requirements (Babe and Craik, 1997; Tong, 2002; Verdaguer et al., 2014). This stringent specificity of viral proteases makes them successful targets as biotechnological tools (Kim et al., 2012; Fernandez-Rodriguez and Voigt, 2016; Tran et al., 2017) and for antiviral therapies (Shamsi et al., 2016). Different drugs targeting proteases have been used effectively for treating animal viral infections (Anderson et al., 2009; Clark et al., 2013; Lv et al., 2015), and have also had moderate success in the plant world (García et al., 1993; Gutiérrez-Campos et al., 1999, 2001; Wen et al., 2004; Gholizadeh et al., 2005; Habib and Fazili, 2007; Kim et al., 2016).

**Table 1:** Plant viral proteases.

Family	Group^1^	Proteases	MEROPS^2^ family	Type/fold	Action	Suggested specific function
*Potyviridae*	(+)RNA Picorna-like	P1a-like	S30	Serine/ Chymotrypsin-like	*Cis*	Replication Counterdefense Host range
		P1b-like	S30	Serine Chymotrypsin -like	*Cis*	Counterdefense
		HC	C6	Cysteine/ Papain-like	*Cis*	Aphid transmission Counterdefense Virion assembly
		NIapro	C4	Cysteine/ Chymotrypsin -like	*Cis/trans*	Replication Host range Superinfection exclusion
		P2-1	C6	Cysteine/ Papain-like	*Cis*	Unknown
*Secoviridae*	(+)RNA Picorna-like	Pro	C3	Cysteine/ Chymotrypsin -like	*Cis/trans*	Replication Counterdefense
*Luteoviridae*	(+)RNA Picorna-like	Protease	S39	Serine/ Chymotrypsin-like	*Cis*/*trans*	Replication
*Solemoviridae*	(+)RNA Picorna-like	P1	Unclassified	Unknown	*Cis*	Counterdefense
		Pro	S39	Serine/ Chymotrypsin-like	*Cis*/*trans*	Replication
*Tymoviridae*	(+)RNA Alpha-like	PRO	C21	Cysteine/ Papain-like	*Cis*/*trans*	Replication Counterdefense
*Closteroviridae*	(+)RNA Alpha-like	L/P/L1/L2	C42	Cysteine/ Papain-like	*Cis*	Systemic movement Host range Superinfection exclusion
*Betaflexiviridae*	(+)RNA Alpha-like	PRO	C23	Cysteine/ Papain-like	*Cis*/*trans*	Replication
*Benyiviridae*	(+)RNA Alpha-like	PCP	C36	Cysteine/ Papain-like	*Cis*/*trans*	Replication Counterdefense
*Endornaviridae*	dsRNA Alpha-like	CRR?	Unclassified	Cysteine?	*Cis*/*trans*?	Unknown
*Pseudoviridae*	(+)RNA Retrovirus	PR	A11	Aspartic/ Pepsin-like	*Cis*/*trans*	Virion maturation?
*Metaviridae*	(+)RNA Retrovirus	PR	A2	Aspartic/ Pepsin-like	*Cis*/*trans*	Virion maturation?
*Caulimoviridae*	dsDNA Pararetrovirus	PR	A3	Aspartic/ Pepsin-like	*Cis*/*trans*	Virion maturation

*^*1*^Classification of RNA viruses based on Dolja and Koonin (2011).*

*^*2*^MEROPS classification of proteases (Rawlings et al., 2013).*

Plant viral proteases carry out multiple roles during viral infection independent of their protease activity: RNA silencing suppression, aphid transmission, systemic transport, viral accumulation, viral particle maturation, etc. (Liu et al., 2009; Csorba et al., 2015; Valli et al., 2018). As proteases, however, the primary role they play in viral infection is processing of and from viral polyproteins. But there is more to this protease activity than just acting as peptide cutters. Polyprotein processing is not an all-or-nothing process in which all products are separated at the same time with perfect efficiency. Cleavage of the polyprotein into functional units is essential for viral survival and it is a highly modulated process. Its regulation modifies the time and place of the final products as well as the possible accumulation of intermediate products, which can play distinct roles in the life cycle. In addition, processing of host proteins can also alter the function of these viral proteases. In general, plant viral proteases have been understudied when compared to their animal counterparts in terms of processing regulation and structure, probably due to the relevant role that the latter play in human health. Nonetheless, a large amount of information has been published about plant viral proteases in recent years leading us to write a review on the subject. This review will give an overview of different roles that lie behind the proteolytic activity of plant virus proteases and emphasize their relevance during viral infection.

## Replication

Key to viral infection is genome replication. It takes place at specific sites in the cell, compartments termed viral factories in which multiple viral and plant factors required for replication are concentrated (Heinlein, 2015). Involvement of viral proteases in these factories has been demonstrated for some cases, but information is not always available. For animal viruses, the role that endopeptidases play in regulating replication is well-established (Sawicki and Sawicki, 1994; Racaniello, 2001; Vasiljeva et al., 2003; Yost and Marcotrigiano, 2013; Rausalu et al., 2016). Information is scarcer in the case of plant infecting viruses.

The *Potyviridae* is a family of positive-stranded RNA viruses that belongs to the picornavirus-like supergroup. It comprises 10 genera and presents the highest protease variety among plant viruses, coding in their genomes up to five different proteases with varied specificities [P1 (P1a- and P1b-like), HC, NIapro, P2-1 (HC-like)] (Adams et al., 2005a,b; Rodamilans et al., 2013; Revers and García, 2015). One hallmark of the picorna-like viruses, other than a conserved RdRp, is the presence of a 3C-like protease in charge of polyprotein processing. For the *Potyviridae*, this is NIapro. Indeed, this is the best characterized plant viral protease, functionally and structurally, which modulates replication by polyprotein processing (Carrington and Dougherty, 1987). NIapro is a chymotrypsin-like cysteine protease that acts *in cis* and *in trans* and it is involved in the generation of intermediate (such as P3-6K1, CI-6K2, and 6K2-NIa) and final products at different stages of infection. These products are implicated in the formation of the replication complex and its anchoring to, and release from, ER-derived membranes (Restrepo-Hartwig and Carrington, 1994; Riechmann et al., 1995; Schaad et al., 1997; Merits et al., 2002; Beauchemin et al., 2007; García et al., 2014; Cui and Wang, 2016).

The *Secoviridae* (Thompson et al., 2014), *Luteoviridae* (Prüfer et al., 1999; Li et al., 2000), and *Solemoviridae* families (Satheshkumar et al., 2004; Sõmera et al., 2015) belong to the picornavirus-like supergroup and share equivalent proteases. In the *Secoviridae* family, studies with the waikavirus rice tungro spherical virus (RTSV) (Thole and Hull, 1998), the nepovirus tomato ringspot virus (TomRSV) (Wang et al., 1999; Wang and Sanfaçon, 2000) and strawberry mottle virus (SMoV) (Mann et al., 2017), have characterized the viral protease (Pro) and their cleavage sites, but so far little is known about the specific involvement of these proteases in viral replication. The same is true for the *Luteoviridae* family. The serine protease encoded by the ORF1 of the polerovirus potato leafroll virus (PLRV) is able to act *in cis* and *in trans* and to separate the membrane anchoring portion, the protease and the genome-linked viral protein (VPg) domains; whether this is part of a regulatory mechanism for viral replication is still unknown (Li et al., 2007). Viruses of the *Solemoviridae* family express two versions of a polyprotein, from ORF2a and ORF2b, having different C terminus. The N-terminal common part includes a membrane anchor domain, the protease Pro, and VPg. Polyprotein 2a (P2a) C-terminal part codes for P10 and P8 proteins. Polyprotein 2ab (P2ab) codes for RdRp and is originated by ribosomal frameshift. Studies with sesbania mosaic virus (SeMV) indicate that the serine protease performs differently in P2a and in P2ab (Nair and Savithri, 2010a,b). In the first case, processing occurs at the predicted sites separating all components from the polyprotein. However, in the latter case, processing of VPg from RdRp is not fulfilled even though the protease and cleavage sequence are conserved in P2a and P2ab. This points to a regulatory process in protease activity that might have an influence in replication considering the inhibitory effect observed *in vitro* that VPg has over the polymerase when present at its N terminus. In addition, mutational analysis of cleavage sites indicated that all sites at P2a/P2ab are essential for viral replication, and the products are only functional when released at the site of replication (Govind et al., 2012) reinforcing the modulatory role of the protease.

Another example of plant viral protease involved in replication comes from the *Tymoviridae* family that belongs to the alphavirus-like supergroup. Turnip yellow mosaic viru*s* (TYMV) encodes a papain-like cysteine protease, termed PRO (Rozanov et al., 1995; Lombardi et al., 2013). Involvement of PRO in replication comes from two different lines of evidence: (i) the processing ability of the protease to act *in cis* and *in trans* similarly to the proteases of rubiviruses and alphaviruses, which share a similar polyprotein structure (Jakubiec et al., 2004, 2007) and (ii) its deubiquitination activity (Camborde et al., 2010; Chenon et al., 2012). TYMV is the type member of the genus *Tymovirus*, a single positive-stranded RNA spherical virus that produces two overlapping ORFs from a single RNA. One of them encodes a polyprotein of 206 KDa that contains sequence domains of methyltransferase (MET), PRO, helicase (HEL) and RdRp. PRO was shown not only to separate RdRp from the rest of the polyprotein, but also process HEL in a secondary event. This and the ability of PRO to act *in trans* appear to reflect the evolutionary relationship of this virus to rubiviruses and alphaviruses, and as it occurs in these animal viruses, it is likely that temporal regulation of polyprotein processing controls the synthesis of different RNA species (negative- and positive-strands). Whether the specific cleavage observed in TYMV also shuts off the synthesis of negative-strand RNA is still unknown (Jakubiec et al., 2007). In addition to this, TYMV PRO is a functional ovarian tumor-like deubiquitylating enzyme (DUB) and this activity helps PRO to modulate viral replication by stabilizing the viral polymerase preventing degradation by the ubiquitin-proteosome system (Camborde et al., 2010; Chenon et al., 2012; Bailey-Elkin et al., 2014; Jupin et al., 2017).

The alphavirus-like supergroup does not maintain a conserved protease in all members as the picornavirus-like does. In this way, the *Closteroviridae* family, although sharing in ORF1a the MET, HEL organization followed by RdRp in ORF1b, does not encode a protease that acts *in trans* to process these products, but contains a leader proteinase(s) with autocatalytic activity (Dolja et al., 2006; Agranovsky, 2016). On the other hand, some members of the *Betaflexiviridae* family, do encode in ORF1 similar MET, PRO, HEL, RdRp domains as members of the *Tymoviridae* family do, although there is little information regarding polyprotein processing and no data regarding involvement of PRO in replication (Foster and Mills, 1992; Lawrence et al., 1995). A similar lack of information is encountered in the *Benyiviridae* family. Its most studied member, beet necrotic yellow vein virus (BNYVV), encodes a papain-like cysteine protease domain (PCP) (Hehn et al., 1997), and it has been hypothesized that it might act as a DUB to favor RdRp transcription (Pakdel et al., 2015), similar to the mode of action of the PCP domain of hepatitis E virus (HEV), although in the latter case, PCP acted as a DUB to counteract cellular antiviral pathways (Karpe and Lole, 2011).

But not only *trans*-acting proteases are involved in the regulation of replication. Recently, the leader protease P1 of the *Potyvirus* genus has also been assigned to this role. Work performed with plum pox virus (PPV) P1 showed that the N-terminal part of this *cis*-acting serine proteinase, the most variable region, acts as a negative regulator of P1 self-processing, modulating in this way potyviral replication (Pasin et al., 2014). Removal of the N-terminal part of P1, not only makes the protein co-factor independent, but also potentiates viral replication at early times of infection emphasizing the regulatory role of this protein in the potyviral life cycle. The way PPV P1 is modulating replication through host factor interactions resembles the mode of action of the NS2 protease of animal virus bovine viral diarrhea virus (BVDV) (Lackner et al., 2004, 2006). In this pestivirus, the NS2 protease modulates replication indirectly by downregulating NS2-NS3 processing. Similarly, PPV P1 modulates P1HC processing and indirectly affects viral replication.

## Viral Counterdefense

Sometimes, when a protease potentiates a positive effect on replication it is not due to a specific role in this viral process, but it is the consequence of an indirect effect caused by an enhanced ability of the virus to escape plant defenses. Thus, proteases could be considered as having a counterdefense role instead of a role in viral replication. For example, PPV P1 was described as having a modulatory role in replication, but this is likely derived from the modulation of the RNA silencing suppressor HC. It can be considered that P1 is actually modulating host defense responses and that the effect observed in viral replication is just a by-product of this role. The same can be argued in the case of TYMV PRO activity as DUB, which can be viewed not in terms of modulating replication, but if RdRp degradation is considered as part of plant defense, it can be viewed as a counterdefense mechanism (Camborde et al., 2010; Chenon et al., 2012; Lombardi et al., 2013; Jupin et al., 2017).

Using DUBs as a means of protection against host defenses is something well-established in the animal viral world. Examples can be found among viruses of the order *Nidovirales* such as the coronavirus severe acute respiratory syndrome-related coronavirus (SARS-CoV) or the arterivirus equine arteritis virus (EAV) that use this strategy of interfering with the innate immune signaling pathway through the DUB activity of their cysteine proteases (Clementz et al., 2010; van Kasteren et al., 2013). The same is true for viruses of the order *Picornavirales* such as the aphtovirus foot-and-mouth disease virus (FMDV) and its L^pro^ leader protease (Wang et al., 2011a,b). In all these cases, however, although the counterdefense activity is well-documented, it appears that the DUB and the protease activity are not strictly interrelated. In the case of TYMV, these two activities can be uncoupled by mutations that selectively suppress the DUB activity without altering PRO (Jupin et al., 2017).

Probably, the best characterized proteases acting as viral counterdefense barriers by degrading host proteins are the ones from the *Picornaviridae* family (Agol and Gmyl, 2010). Thus, FMDV L^pro^ not only disrupts the interferon signaling pathway through its deubiquitinase activity but also cleaves eIF4G shutting off host cap-dependent translation and downregulating Type I interferons (Guarné et al., 1998; Chase and Semler, 2012; Liu et al., 2015). Moreover, FMDV produces, as the rest of the members of the *Picornaviridae* family, 3C^pro^, a protease that is in charge of processing the different elements of the polyprotein acting *in cis* and *in trans*, and also degrades several host proteins in order to potentiate viral transcription and translation (Sun et al., 2016). In the same family, rhinoviruses and enteroviruses produce another protease termed 2A^pro^, which also develops these degrading functions (Seipelt et al., 1999; Chase and Semler, 2012).

Taking these activities into account it is reasonable to ask the question of whether the 3C-like proteases of plant picorna-like viruses perform similar host degrading activities to counteract plant defenses or not. In the case of NIapro, the 3C-like protease of the *Potyviridae*, no specific host proteins affected by its catalytic activity have been described and, only recently, a study was published describing possible interacting partners in plants (Martínez et al., 2016). However, it cannot be ruled out that NIapro might be processing more proteins than the viral ones taking into consideration its demonstrated ability to act on proteins with an engineered target sequence (Rohila et al., 2004; Cesaratto et al., 2016) or even on proteins with a naturally occurring target cleavage site, such as the amyloid-β peptide (Han et al., 2010; Kim et al., 2012). Likewise, NIapro from potato virus Y (PVY) acts as elicitor of the hypersensitive response mediated by the gene *Ry* in potato, and its protease activity, likely acting on a host factor, appears to be involved in this eliciting response (Mestre et al., 2000, 2003). More recent studies have described a role of potyviral NIapro in enhancing aphid transmission and suggested that this role might be related to its ability to degrade vacuolar defense proteins (Casteel et al., 2014; Bak et al., 2017).

Some newly published reports add more information to the scarce available data about activities of 3C-like proteases related with defense and counterdefense responses. The RNA silencing suppressor R78 of the waikavirus maize chlorotic dwarf virus (MCDV) is cleaved by Pro, raising the possibility that this cleavage might have some influence in R78 silencing suppression activity over the course of the infection (Stewart et al., 2017). Moreover, NIapro of the tritimovirus wheat streak mosaic virus (WSMV) contributes to prevent superinfection by related viruses, and it has been suggested that the protease activity of this protein is required for superinfection exclusion (Tatineni and French, 2016).

## Virion Maturation

A good example of a viral protease directly involved in virion formation is togavirin from viruses of the genus *Alphavirus*. Structurally related to chymotrypsin-like serine proteases, togavirin is the actual core protein. It self-processes from the polyprotein precursor, binds viral RNA, and assembles into the capsid (Krupovic and Koonin, 2017). Apart from this versatile endopeptidase, the role of proteases in virion maturation has been well-studied for animal retroviruses such as human immunodeficiency virus (HIV), Rous sarcoma virus (RSV) or murine leukemia virus (MLV), amongst others (Konvalinka et al., 2015). In these viruses, cleavage of viral polyproteins at specific sites and in an orderly fashion is crucial for transforming the immature shell into an active infectious particle. *Pseudoviridae* and *Metaviridae* are two viral families that include plant retroviruses (Peterson-Burch and Voytas, 2002; Wright and Voytas, 2002; Eickbush and Jamburuthugoda, 2008), but there is not much information regarding the regulation of proteolytic processing. More data is available about the *Caulimoviridae*, the single family of plant pararetroviruses (Torruella et al., 1989). The genome of all replication-competent retroviruses consists of structural, replication and envelope proteins (gag, pol, and env) (Marmey et al., 2005). The protease (PR), an aspartate peptidase with no homology to other viral proteases, is generally included in the pol domain. Viruses of the *Caulimoviridae* family, the only plant viruses with dsDNA genomes, encode the gag-pol core, but unlike retroviruses, lack an integrase, which is not required because the caulimoviral DNA is not integrated in the host chromosome. The type virus of the family is cauliflower mosaic virus (CaMV), a member of the *Caulimovirus* genus. The capsid protein (CP) of this virus is produced as a precursor (pre-CP) with N- and C-terminal extensions. CP is involved in virion assembly, packaging of viral RNA and delivery of the genome to the nucleus. Processing of the CP extensions is thought to regulate these functions. The N-terminal extension of CP appears to be involved in keeping the pre-CP in the cytoplasm and may operate as an anchoring domain for the initiation of viral assembly, similar to what occurs to HIV viral matrix protein (Champagne et al., 2004). Virion maturation is completed by removal of the first 76 aa and about 40 aa from the C terminus by the viral aspartic proteinase (Karsies et al., 2002; Champagne et al., 2004). The fact that pre-CP is excluded from the nucleus, would assure that only mature virions, containing the genomic DNA, enter in the nucleus (Karsies et al., 2002). Studies done with another plant pararetrovirus, the badnavirus rice tungro bacilliform virus (RTBV), showed that its aspartic protease cuts independently of plant-specific host factors since it retained its proteolytic activity in baculovirus (Laco et al., 1995) and bacteria (Marmey et al., 2005). In the case of animal retroviruses, PR is expressed in an inactive monomeric form and needs to dimerize to acquire an active conformation in which each unit contributes an aspartate to the active site. Proper redox environment is likely to also play a role in PR activation (Ingr et al., 2003; Konvalinka et al., 2015). Based on active site comparison, it is anticipated that PR of *Caulimoviridae* also acts as dimers (Torruella et al., 1989). Its activation requirements are still pending further investigation.

## Host Range Definition

Plant viruses have definite host ranges, which in some cases are very narrow. The complex network of interactions between plant and virus that needs to be established in order for the infection to progress makes it difficult for the virus to have broad host spectrum. In terms of viral proteases, the best examples of host range modulation come from the *Potyviridae* family (Adams et al., 2005a,b; Revers and García, 2015). Potyviruses, rymoviruses, and some ipomoviruses have P1a-like leader serine proteases whose cleavage is essential for virus infectivity (Verchot and Carrington, 1995). These proteases rely on a plant factor(s) to develop their proteolytic activity, a feature that separates them from P1b-like serine proteases in the family and whose cleavage is co-factor independent (Verchot et al., 1992; Valli et al., 2006; Rodamilans et al., 2013). The comparison of two PPV isolates, which differed in their reciprocal capacity of infecting woody and herbaceous hosts, showed the relevance of P1 among other viral proteins for host adaptation (Salvador et al., 2008a). Similarly, analyses of PPV chimeras including P1 sequences of tobacco vein mottling virus (TVMV) and of virus variants with different biological properties sorted from a single PPV isolate also pointed toward the involvement of P1 in host range definition (Salvador et al., 2008b; Maliogka et al., 2012). All these works show how relevant P1 is in terms of host spectrum characterization, but do not necessarily implicate the protease activity of P1 in this role. More direct evidence of the involvement of P1-mediated proteolytic processing in compatibility with the host comes from works performed with P1a of cucumber vein yellowing virus (CVYV) and P1 of PPV, both P1a-like proteases. In these studies, it was shown that one of the factors limiting PPV infection in *Cucumis sativus* was likely the incompatibility of PPV P1 with a host co-factor required for its protease activity. Either replacing P1 with P1a, supposedly compatible with a cucumber co-factor, or with a host factor-independent P1 mutant, provided PPV the ability to partially break the non-host resistance of cucumber (Carbonell et al., 2012; Shan et al., 2015, 2017).

From the same *Potyviridae* family, NIapro has also been described to play a role in host range determination. In the papaya ringspot virus (PRSV), a single amino acid substitution in this chymotrypsin-like protease allows a host-shift from cucurbits to papaya, although the specific involvement of the protease activity of NIapro in this effect is only a possibility (Chen et al., 2008). More direct evidence of the involvement of the protease activity of NIapro in host range determination comes from work performed with PPV (Calvo et al., 2014). This study showed that alternative adaptation to *Nicotiana* and *Prunus* hosts was determined, not by peculiarities of the NIapro sequence, but by differences in the NIapro target sequence placed between 6K1 and CI, suggesting modulation of NIapro processing at this site in a host-specific manner.

## Proteolytic Activity-Unrelated Functions

The small size of the genome of plant RNA viruses forces the proteins from these viruses to acquire multiple functions. This is best exemplified by the potyviral protein HC (Valli et al., 2018). HC is a cysteine proteinase whose first identified function was to aid in aphid transmission of viral particles (de Mejia et al., 1985). However, the main function of the potyviral HC appears to be suppressing antiviral RNA silencing (Anandalakshmi et al., 1998; Kasschau and Carrington, 1998), and an independent function of HC in the correct assembly of potyviral virions has been more recently reported (Valli et al., 2014). Interestingly, all these HC functions do not rely on its proteolytic activity, as it is also the case for the RNA silencing suppression activity of the serine proteinase P1b of the ipomovirus CVYV (Valli et al., 2008), illustrating how proteolysis-related and -unrelated roles can concur in a single viral protein. Probably also unrelated to its protease activity is the role suggested for P1 of tobacco etch virus (TEV) in stimulating viral RNA translation (Martínez and Daròs, 2014).

Viral proteinases with functions that appear unrelated to their proteolytic activity are not restricted to the family *Potyviridae*. The self-cleaving leader proteinases of viruses of the *Closteroviridae* family are a good example of this. These proteinases are involved in virus accumulation, systemic transport, host range expansion or virus superinfection exclusion, but all these roles appeared to be independent of their protease activities (Peng et al., 2001, 2003; Liu et al., 2009; Atallah et al., 2016). Contrary to what was observed for the leader proteinase of FMDV, the closterovirus proteases show no DUB activity and have not been described to be involved in further processing of host or viral proteins.

## Concluding Remarks

It is well-established that viral proteases are not just proteolytic machines acting without proper modulation of time and/or space. Much effort has been put into defining what these extra roles are and characterizing the different mechanisms of action and their peculiarities. Involved in regulating replication, virion maturation, host range determination or even displaying a more active role as viral counterdefense barriers, proteases, when present, are essential in practically all aspects of the viral cycle. However, there are still many proteinases from plant viruses for which information about the integration of its enzymatic activity in the infection process is still unavailable. Viruses of the family *Endornaviridae* are a fine example. These viruses have been understudied probably because they do not usually cause any noticeable damage on their hosts. They have a monocistronic RNA genome that encodes a large polyprotein, but there are only hints about how this polyprotein is processed (Roossinck et al., 2011; Sabanadzovic et al., 2016). The case of P1 of the sobemovirus rice yellow mottle virus (RYMV) is another good example of a viral protease with a puzzling role (Weinheimer et al., 2010). RYMV P1, a protein with RNA silencing suppression activity, is expressed as a mature protein, rather than as part of a protein precursor; however, in experimental conditions it displays self-cleaving activity able to precisely remove engineered C-terminal extensions. Maintaining a function that seems to be superfluous raises the possibility that this protease, and by similarity other leader proteases, might have an extra unknown biological function.

We have focused this short review on the roles of virus-encoded proteinases in viral infection. However, control of gene expression by proteolytic processing of protein precursors not only relies on viral proteinases. For instance, host aspartyl proteases are in charge of the processing of the primary product of the M genomic RNA of plant viruses of the order *Bunyavirales* to yield two mature glycoproteins (Whitfield et al., 2005; Li et al., 2015; Shi et al., 2016). The involvement of cellular proteases in modulating plant virus infection is another exciting target for future research.

## Author Contributions

BR drafted the manuscript with the collaboration of JAG. HS and FP contributed to the conception and design of the review and revised the manuscript. All the authors read, critiqued, and approved the final manuscript.

## Conflict of Interest Statement

The authors declare that the research was conducted in the absence of any commercial or financial relationships that could be construed as a potential conflict of interest.
